# What is the actual relationship between neutrophil extracellular traps and COVID-19 severity? A longitudinal study

**DOI:** 10.1186/s12931-023-02650-9

**Published:** 2024-01-19

**Authors:** Cristina de Diego, Ana Belén Lasierra, Lucía López-Vergara, Laura Torralba, Pablo Ruiz de Gopegui, Raquel Lahoz, Claudia Abadía, Javier Godino, Alberto Cebollada, Beatriz Jimeno, Carlota Bello, Antonio Tejada, Salvador Bello

**Affiliations:** 1 Department of Pulmonary Medicine, Miguel Servet University Hospital, CIBERES, Instituto de Investigación Sanitaria (ISS) Aragón, Avenida Isabel la Católica 1-9, 50009 Zaragoza, Spain; 2 Department of Biochemistry, San Jorge Hospital, Huesca, Spain; 3 Intensive Care Unit, Miguel Servet University Hospital, Zaragoza, Spain; 4 Department of Biochemistry. Miguel, Servet University Hospital, Zaragoza, Spain; 5 Department of Cytometry and Cell Separation, Aragon Institute of Health Sciences (IACS), Zaragoza, Spain; 6 Biocomputing Technical Scientific Service, Aragon Institute of Health Sciences (IACS), Zaragoza, Spain; 7 Department of Radiology, Hospital Clínico Lozano Blesa, Zaragoza, Spain

**Keywords:** COVID-19, Neutrophil extracellular traps, NET markers, Longitudinal study, Severity, Mortality

## Abstract

**Background:**

Neutrophil extracellular traps (NETs) have repeatedly been related to COVID-19 severity and mortality. However, there is no consensus on their quantification, and there are scarce data on their evolution during the disease. We studied circulating NET markers in patients with COVID-19 throughout their hospitalization.

**Methods:**

We prospectively included 93 patients (201 blood samples), evaluating the disease severity in 3 evolutionary phases (viral, early, and late inflammation). Of these, 72 had 180 samples in various phases. We also evaluated 55 controls with similar age, sex and comorbidities. We measured 4 NET markers in serum: cfDNA, CitH3, and MPO-DNA and NE-DNA complexes; as well as neutrophil-related cytokines IL-8 and G-CSF.

**Results:**

The COVID-19 group had higher CitH3 (28.29 *vs* 20.29 pg/mL, p = 0.022), and cfDNA, MPO-DNA, and NE-DNA (7.87 vs 2.56 ng/mL; 0.80 vs 0.52 and 1.04 vs 0.72, respectively, p < 0.001 for all) than the controls throughout hospitalisation. cfDNA was the only NET marker clearly related to severity, and it remained higher in non-survivors during the 3 phases. Only cfDNA was an independent risk factor for mortality and need for intensive care. Neutrophil count, IL-8, and G-CSF were significantly related to severity. MPO-DNA and NE-DNA showed significant correlations (r: 0.483, p < 0.001), including all 3 phases and across all severity grades, and they only remained significantly higher on days 10–16 of evolution in those who died. Correlations among the other NET markers were lower than expected.

**Conclusions:**

The circulating biomarkers of NETs were present in patients with COVID-19 throughout hospitalization. cfDNA was associated with severity and mortality, but the three other markers showed little or no association with these outcomes. Neutrophil activity and neutrophil count were also associated with severity. MPO-DNA and NE-DNA better reflected NET formation. cfDNA appeared to be more associated with overall tissue damage; previous widespread use of this marker could have overestimated the relationship between NETs and severity. Currently, there are limitations to accurate NET markers measurement that make it difficult to assess its true role in COVID-19 pathogenesis.

**Supplementary Information:**

The online version contains supplementary material available at 10.1186/s12931-023-02650-9.

## Summary at a Glance

Circulating markers evidenced NETs formation by Covid-19 throughout hospitalization and in all grades of severity, but showed limited relationship with severity and mortality, and different degrees of specificity, making it difficult to assess their true role in the disease outcomes.

## Background

Elevated neutrophil levels are early indicators of severe acute respiratory syndrome coronavirus 2 (SARS-CoV-2) infection and severe disease [1–5]. Their effector functions are multiple, including enzyme degranulation, pathogen destruction by reactive oxygen species (ROS), phagocytosis, and neutrophil extracellular traps (NETs). NETs are networks of chromatin, intranuclear proteins (histones), and granule enzymes present in extracellular medium after neutrophilic destruction, to further control bacterial, fungal, viral, or parasitic infection [6]. This cell death mechanism, or NETosis, is distinct from apoptosis and necrosis, with an alternative mechanism, exocytosis of granules and nuclear material, termed vital or non-lytic NETosis [7, 8]. Peptidylarginine deiminase (PAD4) activation results in histone citrullination [9], although it is not known whether citrullination is a cause or a consequence of histone externalization. All these products, in cases of excessive production and/or reduced clearance [10, 11], can cause significant tissue damage. NETosis triggers are multiple, including virus-damaged epithelial cells, activated platelets and endothelial cells, and inflammatory cytokines, including interleukin (IL)-1, IL-8, and granulocyte colony-stimulating factor (GCSF) [2]. During the COVID-19 pandemic, certain NET components, such as cell-free DNA (cfDNA) and extracellular histones, have been shown to be damage-associated molecular patterns (DAMPs) from damaged cells, which regardless of their origin have been involved in its pathogenesis [12]. The presence of potential circulating NET markers [2, 4, 5, 12–14], in respiratory samples [13, 15] or in tissues [5, 15–18] has been associated with severity and mortality.

NET quantification typically assesses the simultaneous presence of its components as biomarkers, including cfDNA, histones (H3) or citrullinated histones (CitH3), myeloperoxidase (MPO) isolated or in complexes with DNA (MPO-DNA), neutrophil elastase (NE), and NE-DNA complexes. Those considered more specific are those less likely to originate from activation and death of neutrophils other than NETs, such as from other cells or damaged tissues, or incidentally from molecular interactions in plasma. The choice of markers is a potential source of contradictory conclusions, and few studies exist in longitudinal cohorts to improve knowledge of their predictive power. After having demonstrated the usefulness of cfDNA as a prognostic marker in patients hospitalised for COVID-19 [19], we aimed to check whether these elevated figures were related to NETs, studying the most specific NET markers longitudinally and throughout the hospitalization of our patient group.

Since the beginning of the pandemic, the disease progression time has been considered important for prognostic and therapeutic measures. From the onset of symptoms, there is an initial viral replication and elimination phase lasting approximately 7 days, followed by a decrease in viral presence and increased inflammation (early inflammation phase). In the case of progression, there is disbalance of the immune response by day 16 and later (late inflammation phase), and potentially life-threatening pulmonary and systemic alterations can occur [20]. Our objective was to study the evolution of circulating markers of neutrophilic activation and NETs, including those considered to have high specificity [21], throughout the hospitalization of patients with Covid-19 and their relationship with severity and mortality.

Our hypotheses:

1. NETs markers are increased in patients with COVID-19 throughout their hospital evolution.
2. These markers are associated with severity or mortality at some point in the evolution of hospitalised patients with COVID-19.

## Methods

### Patients and controls

Patients with COVID-19 hospitalised in April and May 2020 were prospectively enrolled. Exclusion criteria were refusal to sign informed consent, concomitant infection during hospitalization, and immunosuppression (transplant recipients, hematologic malignancies, chemotherapy, or prednisone equivalent ≥ 20 mg/day). The patients were grouped according to severity, based on the Chinese Center for Disease Control and Prevention (CCDC) classification [22] and the World Health Organization Ordinal Scale (WHO OS) [23] as follows: moderately ill (MI), severely ill (SI), and critically ill (CI). According to the day of symptom onset, 3 phases were considered: viral (1–9 days from symptom onset), early inflammation (10–16 days from symptom onset), and late inflammation (> 16 days from symptom onset).

We selected 55 non-hospitalised controls without any infection, of similar age and chronic pathologies as the patients, all of whom underwent routine laboratory tests. All met the same exclusion criteria as the patient group. To compare the three dependent groups (severity groups or the three disease progression phases), we would need a total sample size of 100 (33 by group).

### Blood samples

The samples were obtained from blood requested for healthcare reasons. Patients with at least 2 blood samples in 2 consecutive phases were included in the longitudinal study. We measured neutrophil count, cytokines related to neutrophil activation and recruitment (IL-8, G-CSF), and 4 NET markers: cfDNA, CitH3, and MPO-DNA and NE-DNA complexes.

### IL-8 and G-CSF

IL-8 and G-CSF in serum were quantified employing a bead-based multiplex immunoassay for R&D (LXSAHM-07) and a LABSCAN 100 (Luminex corporation). Data were analysed with Xponent software.

### NE-DNA and MPO-DNA complexes

Serum samples were analysed by enzyme-linked immunosorbent assay (ELISA) of NE-DNA and MPO-DNA complexes, employing anti-NE and anti-MPO antibodies (07–496-I and MABS461 from Sigma Aldrich) and peroxidase conjugate anti-DNA antibody (Cell Death Detection ELISA Kit 154467500 Roche) as previously described [24].

### Citrullinated histone H3

Serum samples were analysed employing a citrullinated histone H3 (Clone 11D3) ELISA kit (Cayman 501620) for quantification according to the manufacturer’s instructions, and 450-nm ODs were read in a Sinergy HT reader (Biotek).

### Cell-free DNA

DNA was purified following the manufacturer’s procedure (ChargeSwitch gDNA 1 mL Serum Kit), adjusting the reactant quantity to the serum sample volume (100 μL). PicoGreen reagent was added in a 1:1 ratio to the purified sample (previously diluted to 1/5 in TE buffer), and the mix was incubated 5 min at room temperature in the dark (Quant-iT PicoGreen dsDNA Assay Kit, Invitrogen P7589). The Synergy HT reader (Biotek) was used to read the sample’s fluorescence (excitation 485 nm, emission 528 nm), and cfDNA concentrations were calculated with the standard provided in the kit.

### Statistical analysis

According to normal criteria (Shapiro–Wilk test), data were presented as mean (standard deviation) or median (interquartile range) and analyzed with the Mann–Whitney U, Kruskal–Wallis, Student’s, or analysis of variance test, as appropriate. Correlations between biomarkers and clinical variables were analysed with Spearman’s rho correlation. Receiver operating characteristic (ROC) analysis was performed to determine the predictive power of biomarkers for various outcomes. A Wilcoxon signed-rank test was performed for the longitudinal study.

Logistic regression was used for specific outcomes, adjusting for age > 60 years, male sex, hypertension, obesity, diabetes, and corticosteroid therapy. The variables selected for the multivariate model were age, sex, and those with a p-value less than 0.05 in the bivariate analysis. All statistical analyses were performed with R version 4.0.5 (R Core Team 2021), and P-values < 0.05 were considered significant. The study was approved (03/04/2020) by IRB Aragón (Aragon Research Ethics Committee [CEICA]) (07/2020).

## Results

### Patients and controls

We collected 201 samples from 93 patients, 72 of whom provided samples (180) from at least 2 disease progression stages. Of the 180 samples from the 72 patients, 87 corresponded to the viral phase, 53 to the early inflammatory phase and 40 to the late inflammatory phase. Thirty-two patients had three or more samples, and eight patients had samples from all three disease progression phases. The median number of days from symptoms onset for the viral, early inflammatory, and late inflammatory phase samples were 6 (5–8), 12 (11–14), and 20 (18–24), respectively. Twelve patients required admission to the intensive care unit (ICU); 19 died. There were no statistically significant differences in the patients’ (n = 93) and controls’ (n = 55) demographics (age and sex) or comorbidities (hypertension; diabetes; obesity; respiratory, cardiac, renal, or hepatic disease; dementia; dyslipidemia) (p > 0.05 for all) (Additional file 1: Table S1). At admission, 56 of the study 93 patients had moderate disease, 29 had severe disease, and 8 were initially classified as critical. Table 1 presents the patients’ information according to severity and samples at each stage.

**Table 1:** Number of patients in each severity group and number of samples of the patients followed longitudinally, according to time from symptom onset

WHO OS scale	1	2	3	4	5	6	7	8	Total
Patients (n)	0	0	7	41	20	1	5	19	93

CCDC scale	Mild-moderate				Severe		Critical		Total
Patients (n)	51				15		27		93

Illness onset	Viral phase			Early inflammation			Late inflammation		Total
Samples (n) from 93 patients	97			59			45		93
Samples (n) from 72 patients	87			53			40		

*CCDC scale* Chinese Center for Disease Control and Prevention classification, *WHO OS* World Health Organization Ordinal Scale

### NETs markers in patients and controls

**Fig. 1:**
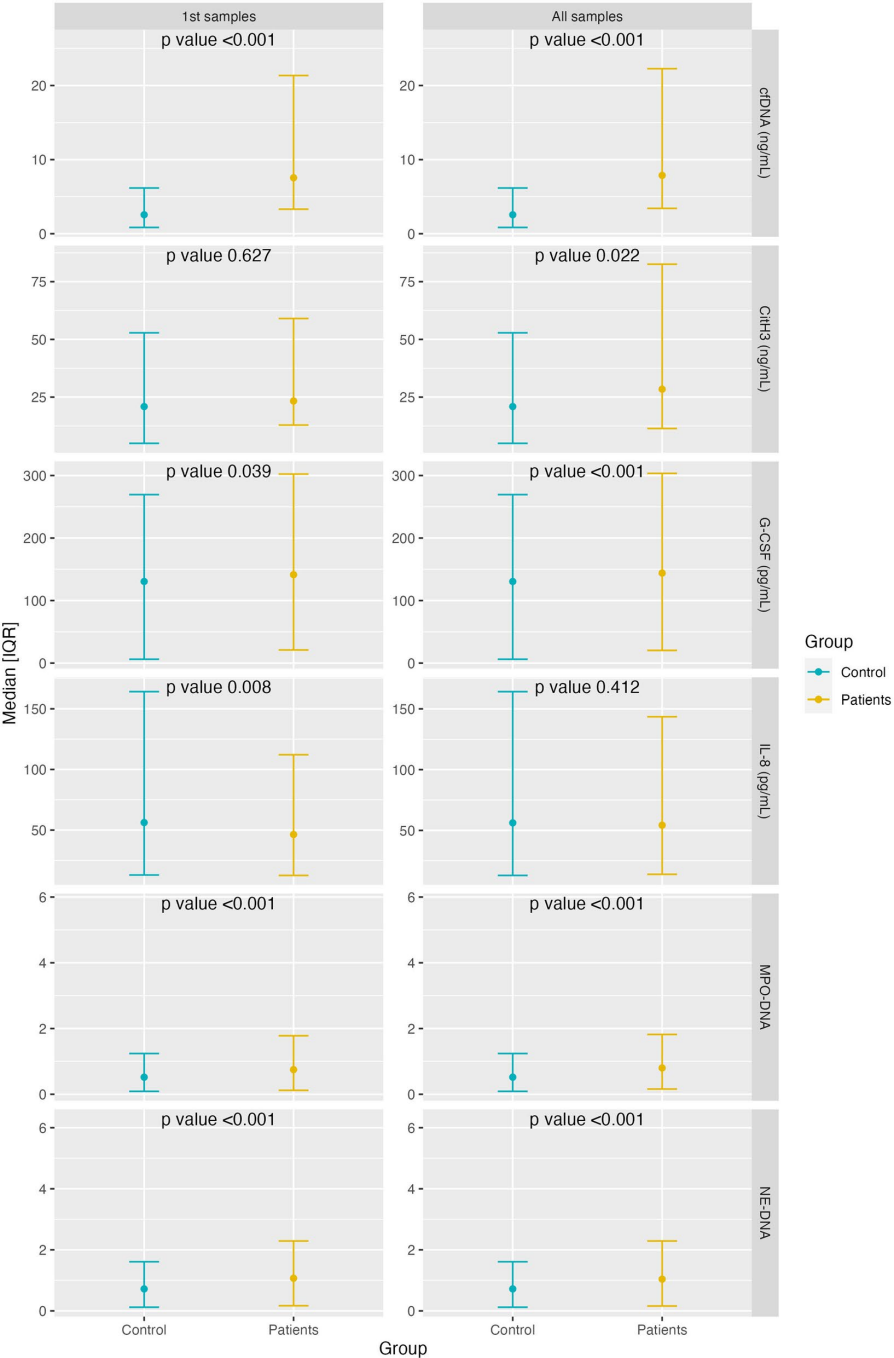
Comparison of biomarkers between patients and controls for all samples and for only the first sample after admission. Median comparison was performed using Wilcoxon signed-rank test

On admission, we observed elevated levels compared with controls in 3 of the 4 NET markers studied: cfDNA and MPO-DNA and NE-DNA complexes (p < 0.001 for all) (Fig. 1). The same occurred in the first 9 days of disease evolution (viral phase), and from days 10–16 (early inflammation phase), in which we also observed elevated CitH3 values (p = 0.003). These differences were maintained from day 17 (late inflammation phase) (p < 0.001 for all 4 markers) (Table 2). Even the MI patients (according to WHO OS) presented increased values in the 4 NET markers: MPO-DNA (p < 0.001), NE-DNA (p < 0.001), CitH3 (p = 0.05), and cfDNA (p < 0.001). In the SI and CI patients together, all 4 NETs markers remained significantly elevated relative to controls (p = 0.041 for CitH3 and p < 0.001 for the others).

**Table 2:** Comparison of biomarkers between patients and controls during the three disease progression phases

Viral phase (1–9 days)	Patients N = 93	Controls N = 55	p-value
IL-8 (pg/mL)	50.31 [34.62; 69.05]	56.21 [43.26; 107.75]	0.025
G-CSF (pg/mL)	138.42 [120.43; 159.45]	130.47 [124.32; 138.97]	0.032
MPO-DNA	0.75 [0.63; 0.95]	0.52 [0.43; 0.72]	< 0.001
NE-DNA	1.06 [0.87; 1.25]	0.72 [0.60; 0.89]	< 0.001
cfDNA (ng/mL)	6.67 [4.04; 13.69]	2.56 [1.71; 3.61]	< 0.001
CitH3 (ng/mL)	23.35 [11.32; 34.99]	20.91 [15.92; 31.94]	0.599

Early inflammation (10–16 days)	Patients N = 59	Controls N = 55	p-value
IL-8 (pg/mL)	55.02 [42.51; 88.89]	56.21 [43.26; 107.75]	0.725
G-CSF (pg/mL)	146.39 [129.14; 162.05]	130.47 [124.32; 138.97]	0.001
MPO-DNA	0.90 [0.64; 1.10]	0.52 [0.43; 0.72]	< 0.001
NE-DNA	1.03 [0.87; 1.25]	0.72 [0.60; 0.89]	< 0.001
cfDNA (ng/mL)	7.51 [5.04; 11.66]	2.56 [1.71; 3.61]	< 0.001
CitH3 (ng/mL)	35.89 [21.49; 68.04]	20.91 [15.92; 31.94]	0.003

Late inflammation (> 16 days)	Patients N = 45	Controls N = 55	p-value
IL-8 (pg/mL)	78.18 [50.36; 111.21]	56.21 [43.26; 107.75]	0.093
G-CSF (pg/mL)	149.48 [132.43;157.64]	130.47 [124.32; 138.97]	< 0.001
MPO-DNA	0.87 [0.74; 1.05]	0.52 [0.43; 0.72]	< 0.001
NE-DNA	1.00 [0.88; 1.32]	0.72 [0.60; 0.89]	< 0.001
cfDNA (ng/mL)	11.24 [5.77; 17.77]	2.56 [1.71;3.61]	< 0.001
CitH3 (ng/mL)	54.54 [28.72; 89.53]	20.91 [15.92; 31.94]	< 0.001

### NET markers, neutrophil count, IL-8, G-CSF, and severity and mortality

Among the 4 NETs markers, we only found significant cfDNA increases in MI vs SI and CI, but not in CitH3, MPO-DNA, or NE-DNA complexes. The same occurred comparing MI and SI vs CI (CCDC) (Table 3). Of the 4 NET markers, only cfDNA showed significant increases in CI vs non-CI (CCDC and WHO OS scales), in the first sample and in the 3 phases of the study. Neutrophil count, IL-8, and G-CSF were significantly related to severity (Table 3). cfDNA was also the single NET marker with higher levels in non-survivors compared with survivors, including in the total sample (p < 0.001), at admission, and in the 3 phases of the disease. The same occurred with neutrophil count. MPO-DNA and NE-DNA showed higher values in non-survivors, but only in the early inflammation phase (Table 4). The non-survivors showed elevated neutrophils, IL-8, and cfDNA in the first sample after admission. There were no differences in CitH3 levels between non-survivors and survivors at any time. In our multivariate model, only cfDNA and obesity were independent risk factors for mortality and need for ICU (Table 5, and Additional file 2: Table S2). The area under the curve (AUC) ROC for critical status and mortality was clearly higher for cfDNA than for the other parameters (Table 6). Analysis examining the relationship between NETs markers and sex, age, and obesity only revealed a statistically significant association between cfDNA and sex (p = 0.002), with higher levels observed in males (14.7 ng/mL) compared to females (7.5 ng/mL). However, this association was not observed in the control group (p = 0.317) (Additional file 3: Table S3). Sex-biased studies were performed for the different outcomes and the same results were obtained regardless of sex (Additional file 4: Table S4).

**Table 3:** Comparisons of biomarkers according to severity (CCDC scale and WHO OS)

	Severe and critical	Moderate	p-value
CCDC scale			
Neutrophils/mm3	6700 [4575; 10725]	4600 [3500; 6400]	< 0.001
IL-8 (pg/mL)	58.22 [46.90; 97.08]	49.89 [35.59; 80.94]	0.015
G-CSF (pg/mL)	148.16 [126.10; 163.97]	142.01 [122.64; 156.55]	0.162
MPO-DNA	0.84 [0.64; 1.03]	0.78 [0.63; 0.99]	0.563
NE-DNA	0.98 [0.86; 1.29]	1.06 [0.91; 1.24]	0.394
cfDNA (ng/mL)	9.44 [6.15; 20.19]	6.21 [3.46; 10.44]	< 0.001
CitH3 (ng/mL)	28.28 [16.19; 57.92]	28.43 [17.93; 51.13]	0.670
WHO OS			
Neutrophils/mm3	6600 [4500; 10775]	4600 [3550; 6350]	< 0.001
IL-8 (pg/mL)	58.80 [46.72; 102.29]	49.15 [36.85; 72.20]	0.007
G-CSF (pg/mL)	150.75 [131.66; 168.10]	136.76 [120.48; 153.66]	0.002
MPO-DNA	0.80 [0.64; 1.02]	0.80 [0.64; 1.00]	0.873
NE-DNA	1.04 [0.87; 1.31]	1.05 [0.89; 1.24]	0.945
cfDNA (ng/mL)	9.08 [5.54; 19.38]	6.88 [3.61; 10.73]	0.001
CitH3 (ng/mL)	31.79 [15.08; 57.53]	26.80 [18.37; 49.48]	0.388

	Critical	Moderate and severe	p-value
CCDC scale			
Neutrophils/mm3	8250 [5400; 12100]	4900 [3700; 6600]	< 0.001
IL-8 (pg/mL)	68.60 [47.40; 94.41]	52.57 [37.56; 82.75]	0.058
G-CSF (pg/mL)	152.80 [131.66; 177.42]	140.17 [122.64; 156.55]	0.009
MPO-DNA	0.84 [0.68; 1.01]	0.78 [0.63; 1.01]	0.470
NE-DNA	0.99 [0.86; 1.35]	1.05 [0.89; 1.24]	0.669
cfDNA (ng/mL)	14.11 [7.13; 22.67]	7.01 [4.01; 10.93]	< 0.001
CitH3 (ng/mL)	27.67 [12.88; 53.68]	30.58 [19.08; 54.70]	0.324

**Table 4:** Comparisons of median biomarker levels between survivors and non-survivors

A			
Total samples (n = 201)	Survivors (n = 158)	Non-survivors (n = 43)	p-value
Neutrophils/mm3	5100 [3700; 6700]	8400 [6050; 13600]	< 0.001
IL-8 (pg/mL)	52.82 [37.84; 83.46]	69.12 [49.89; 104.92]	0.043
G-CSF (pg/mL)	142.57 [122.64; 157.28]	149.55 [130.26; 165.07]	0.090
MPO-DNA	0.80 [0.63; 1.00]	0.80 [0.71; 1.02]	0.517
NE-DNA	1.04 [0.87; 1.23]	1.05 [0.90; 1.48]	0.439
cfDNA (ng/mL)	7.06 [4.12; 11.40]	14.98 [7.42; 25.74]	< 0.001
CitH3 (ng/mL)	28.29 [18.10; 53.97]	29.29 [12.34; 58.58]	0.745

1st sample (n = 93)	Survivors (n = 74)	Non-survivors (n = 19)	p-value
Neutrophils/mm3	5050 [3725; 6550]	8400 [4950; 11750]	0.009
IL-8 (pg/mL)	42.11 [33.18; 56.48]	61.83 [52.88; 89.06]	0.016
G-CSF (pg/mL)	134.22 [118.12; 153.99]	160.09 [139.92; 184.10]	0.012
MPO-DNA	0.75 [0.63; 1.01]	0.77 [0.71; 1.01]	0.508
NE-DNA	1.07 [0.88; 1.19]	1.02 [0.94; 1.57]	0.559
cfDNA (ng/mL)	6.87 [4.01; 10.81]	14.98 [7.73; 22.51]	0.002
CitH3 (ng/mL)	24.51 [12.61; 34.99]	14.80 [6.70; 29.79]	0.129

B			
Viral phase (1–9 days)	Survivors N =78	Non-survivors N =19	p-value
Neutrophils/mm3	4500 [3150; 6600]	7000 [4600; 10550]	0.011
IL-8 (pg/mL)	49.03 [34.24; 61.20]	54.29 [42.30; 74.82]	0.269
G-CSF (pg/mL)	136.45 [118.12; 153.91]	155.74 [128.91; 168.02]	0.069
MPO-DNA	0.72 [0.63; 0.95]	0.75 [0.65; 0.90]	0.988
NE-DNA	1.07 [0.91; 1.23]	0.96 [0.78; 1.34]	0.342
cfDNA (ng/mL)	6.11 [3.48; 11.45]	7.87 [5.08; 22.21]	0.037
CitH3 (ng/mL)	24.51 [13.76; 34.99]	16.19 [7.76; 33.13]	0.295

Early inflammation (10 –16 days)	Survivors N = 48	Non-survivors N = 11	p-value
Neutrophils/mm3	5600 [4275; 7000]	11400 [7650; 14450]	< 0.001
IL-8 (pg/mL)	51.93 [40.97; 81.89]	77.53 [60.24; 110.09]	0.052
G-CSF (pg/mL)	146.64 [129.14; 159.15]	142.57 [125.89; 175.26]	0.731
MPO-DNA	0.85 [0.61; 1.03]	1.61 [0.90; 2.87]	0.026
NE-DNA	0.99 [0.86; 1.14]	1.37 [1.10; 1.68]	0.016
cfDNA (ng/mL)	7.11 [4.46; 9.75]	12.59 [7.88; 26.36]	0.008
CitH3 (ng/mL)	35.89 [21.92; 65.96]	34.49 [17.23; 67.53]	1.000

Late inflammation (>16 days)	Survivors N = 32	Non-survivors N = 13	p-value
Neutrophils/mm3	5250 [4075; 6625]	8400.00 [6500.00; 16400.00]	0.001
IL-8 (pg/mL)	79.03 [49.63; 110.03]	75.08 [51.72; 107.04]	0.966
G-CSF (pg/mL)	149.55 [135.01; 156.55]	149.42 [131.66; 161.75]	0.920
MPO-DNA	0.87 [0.77; 1.05]	0.78 [0.69; 1.02]	0.466
NE-DNA	0.99 [0.88; 1.33]	1.06 [0.90; 1.11]	0.548
cfDNA (ng/mL)	8.35 [4.91; 12.94]	24.62 [19.21; 39.06]	< 0.001
CitH3 (ng/mL)	54.13 [29.46; 79.69]	73.28 [34.28; 102.09]	0.520

(MPO-DNA and NE-DNA complex, cfDNA and CitH3) in all samples (n = 201)A: total samples and first sample; B: throughout the 3 evolutionary phases

**Table 5:** Multivariate model for ICU need

ICU admittance	Yes	No	OR univariate	OR multivariate
Age > 60 years n (%)	51 (85.0)	9 (15.0)	1.76 (0.48–8.41, p = 0.421)	0.40 (0.01–9.74, p = 0.599)
Male sex n (%)	34 (85.0)	8 (19.0)	2.76 (0.80–11.06, p = 0.119)	1.84 (0.08–63.05, p = 0.695)
Hypertension n (%)	40 (88.9)	5 (11.1)	0.73 (0.20–2.48, p = 0.619)	–
Obesity n (%)	**7 (53.8**)	**6 (46.2)**	**10.57 (2.69–43.67, p = 0.001)**	**46.21 (3.76–2138.71, p = 0.011)**
Corticosteroid therapy n (%)	36 (76.6)	11 (23.4)	13.75 (2.50–257.24, p = 0.014	19.62 (1.07–2584.67, p = 0.114)
Diabetes n (%)	25 (89.3)	3 (10.7)	0.75 (0.16–2.75, p = 0.680)	–
IL-8 Mean (SD)	71.3 (97.6)	67.9 (32.7)	1.00 (0.99–1.01, p = 0.909)	–
G-CSF Mean (SD)	199.5 (324.7)	170.9 (41.8)	1.00 (0.99–1.00, p = 0.775)	–
cfDNA Mean (SD)	**8.7 (6.5)**	**23.7 (17.4)**	**1.14 (1.06–1.25, p = 0.001)**	**1.22 (1.07–1.53, p = 0.025)**
MPO-DNA Mean (SD)	0.9 (0.6)	1.1 (0.8)	1.57 (0.61–3.46, p = 0.272)	–
NE-DNA Mean (SD)	1.1 (0.4)	1.4 (0.7)	2.49 (0.78–7.95, p = 0.100)	–
CitH3 Mean (SD)	30.1 (25.8)	18.8 (15.3)	0.97 (0.92–1.00, p = 0.174)	–

Bold highlights parameters with significant differences (p < 0.05)

**Table 6:** AUC ROC of NET markers for severity and mortality

Marker	AUC	Threshold	Specificity	Sensitivity
**AUC ROC for critical status**				
cfDNA	0.716 (0.63–0.8)	14.10	0.509	0.818
MPO-DNA	0.535 (0.44–0.63)	0.71	0.740	0.379
NE-DNA	0.520 (0.42–0.62)	0.94	0.431	0.691
CitH3	0.546 (0.45–0.65)	17.14	0.396	0.803
**AUC ROC for mortality**				
cfDNA	0.748 (0.66–0.84)	20.17	0.947	0.450
MPO-DNA	0.465 (0.36–0.57)	0.75	0.579	0.459
NE-DNA	0.540 (0.43–0.65)	1.53	0.899	0.256
CitH3	0.483 (0.37–0.6)	73.18	0.880	0.250

### Evolution of NET markers throughout the disease course

In the longitudinal study, the MPO-DNA and NE-DNA complexes remained elevated and stable during the 3 phases of the disease. cfDNA increased, especially in the late inflammation phase (p = 0.043) compared with the viral phase; levels of CitH3 progressively increased throughout the 3 phases (p < 0.001), doubling between the first and last sample obtained (p < 0.001) (Table 7).

**Table 7:** Comparison of biomarkers among the three disease progression phases

	Viral phase (1) N = 87	Early inflammation (2) N = 53	Late inflammation (3) N = 40	p-value	p-value 1 vs 2	p-value 1 vs 3	p-value 2 vs 3
Neutrophils/mm3	5100 [3350; 6950]	6000 [4400; 8700]	6050 [4100; 8225]	0.069	0.119	0.119	0.874
IL-8 (pg/mL)	52.01 [34.96; 68.72]	55.52 [45.64; 91.01]	77.32 [50.36; 106.06]	0.002	0.048	0.002	0.108
G-CSF (pg/mL)	139.08 [120.98; 160.64]	147.85 [129.84; 162.19]	148.79 [132.43; 157.64]	0.478	0.488	0.488	0.964
MPO-DNA	0.75 [0.62; 0.94]	0.87 [0.63; 1.03]	0.87 [0.72; 1.03]	0.092	0.211	0.112	0.833
NE-DNA	1.05 [0.88; 1.25]	1.03 [0.86; 1.25]	0.97 [0.88; 1.27]	0.909	0.868	0.868	0.868
cfDNA (ng/mL)	6.40 [3.61; 13.99]	7.87 [5.34; 14.65]	10.53 [5.56; 17.30]	0.041	0.200	0.043	0.200
CitH3 (ng/mL)	23.64 [12.73; 35.10]	35.89 [21.92; 61.36]	54.54 [28.72; 91.81]	< 0.001	0.001	< 0.001	0.043

Median comparison was performed using Wilcoxon signed-rank test

### Correlations

There were significant correlations between MPO-DNA and NE-DNA complexes (r = 0.483, p < 0.001), and less correlation between cfDNA and CitH3 (r = 0.277, p < 0.001) and between cfDNA and NE-DNA (r = 0.168, p = 0.022) (Table 8). We found no other correlations among the 4 NET markers. There were significant correlations between MPO-DNA and NE-DNA (r = 0.793, p < 0.001 in MI; r = 0.472, p < 0.001 in SI; and r = 0.627, p < 0.001 in CI [WHO OS]), which remained significant from the sample at hospital arrival (r = 0.586, p < 0.001) and during the 3 phases (r = 0.705, p < 0.001 in viral; r = 0.581, p < 0.001 in early inflammation; and r = 0.476, p = 0.002 in late inflammation). We found no correlation between CitH3 and MPO-DNA or NE-DNA. There were less strong, but significant, correlations between cfDNA and NE-DNA in CI (r = 0.297, p = 0.043) and between cfDNA and CitH3 in CI (r = 0.371, p = 0.009) and SI (r = 0.289, p = 0.004) (WHO OS). In the total sample, cfDNA was the only NET marker that correlated with typical clinical severity factors, such as lymphopenia, neutrophil/lymphocyte ratio, C-reactive protein, lactate dehydrogenase levels, and baseline SaO2 (See Additional file 5: Table S5).

**Table 8:** Correlations between 4 circulating NET biomarkers

Total samples (n = 201)	r	p
cfDNA vs MPO-DNA	0.095	0.203
cfDNA vs NE-DNA	0.168	0.022
cfDNA vs CitH3	0.277	< 0.001
MPO-DNA vs NE-DNA	0.483	< 0.001
MPO-DNA vs CitH3	0.019	0.797
NE-DNA vs CitH3	− 0.010	0.891

## Discussion

The most important results of our study included a clear increase in circulating NET markers in patients with COVID-19 compared with controls, confirming NET formation in hospitalised patients with COVID-19, from admission throughout hospitalization, including less severe cases. cfDNA was the NET marker most strongly associated with severity and mortality, from the first sample and throughout the disease. It was an independent risk factor and showed the best AUC ROC for both outcomes. However, we found less association than expected between the other 3 NET markers and poor outcomes: only increased MPO-DNA and NE-DNA complexes during the second week (days 10–16 from symptom onset) in non-surviving patients. We observed a significant correlation between markers considered highly specific for NETosis: MPO-DNA and NE-DNA complexes, during the entire evolution and at all severity levels, were much higher than that of the other markers among each other. Throughout the evolution, neutrophil count increased with severity and mortality, and IL-8 increased with severity.

The presence of NET biomarkers in patients with COVID-19 has generated interest [2, 4, 5, 12, 18, 25]; most studies have found increased markers in these patients with respect to healthy controls [2, 5, 12, 13, 26]. Increased NET formation from ex vivo neutrophils of patients with COVID-19 [27] and impaired NET degradation in those with COVID-19 have been reported [5, 25].

In our study, every NETosis marker remained elevated from admission (CitH3 from day 10 after the onset of the disease), during the entire hospitalization and at the 3 levels of severity. This, and the high MPO-DNA and NE-DNA correlations in the 3 levels of severity and during the entire evolution, offer little doubt as to the existence of NETosis in hospitalised patients with COVID-19, including in less severe patients. Interestingly, the delayed onset and increased CitH3 suggest that citrullination is a consequence rather than a cause of histone externalization, which is a matter of debate [9]. Other studies have concluded that circulating NET marker levels are related to severity and mortality in COVID-19, and that NETs could play an important role in pathogenesis [2, 4, 5, 12, 14, 15, 26, 28–30], including their identification in fatal disease tissues [5, 30] and the observation of small pulmonary vessel occlusion by NET aggregates [5, 16]. This NETs–severity relationship is the dominant opinion today [11].

However, contradictory results have been observed for the relationship between circulating NET markers and COVID-19 severity [5, 31, 32], viral load [13, 30], or associated obstructive vascular phenomena [5, 13, 16]. Some reasons could include the non-standardized choice of NET markers, which varies between studies (including some less specific ones); an apparent discrepancy between the markers in tissues and in peripheral blood [13]), as well as between serum or plasma [33]; levels can vary significantly with underlying chronic diseases [5]; and some appear to be associated with severity more than others [5]. Furthermore, plasma levels of markers considered specific, such as MPO-DNA appear to be less sensitive in discriminating severity than other NETs measurement methods, such as NETosis induction in plasma samples, as has been recently reported in patients with COVID-19 [32]. In addition, the heterogeneity of the populations, the short half-life of NET markers [34], and the variety of enzymatic pathways triggering NETs [13] can modify the markers and lead to divergent results.

In addition to cfDNA, included in most of the previous studies, we chose 3 NET markers considered the most specific: CitH3, and MPO-DNA and NE-DNA complexes [21]. It is difficult to distinguish NETosis markers from those of neutrophil activation or other types of cell death [35, 36]. cfDNA or nucleosomes (DNA formations with histones or nuclear proteins, such as H3) can also originate from necrotic processes. MPO and NE frequently originate from neutrophil degranulation; however, their binding to DNA (NE-DNA and MPO-DNA complexes used in our study) likely makes them the most specific for NETs, because they are less likely to be formed incidentally by molecular interactions in plasma [21, 35]. Citrullinated histones, such as CitH3, although considered specific, represent only one NETosis pathway (via PAD4) [37]; their levels may be similar in children with COVID-19 as in healthy children [38], and can vary depending on stimulus [39]. A recent study found a much stronger relationship between COVID-19 severity (and long COVID) and increased NETosis-induction capacity measured ex vivo, than that of circulating NET-specific marker levels (MPO-DNA complex) [32].

The lack of correlations observed between some markers considered specific, such as CitH3 with MPO-DNA and NE-DNA, has been reported in COVID-19 between CitH3 and MPO-DNA [2], and between CitH4-DNA and NE-DNA, which showed opposite trends in COVID-19 severity assessment [5]. In another study, CitH3-NE complexes did not differ from those of controls [31]. The simultaneous involvement of alternative non-PDA4 pathway-dependent NETosis in COVID-19 [2, 40] and the existence of sequestered NET fragments in damaged organs could explain the discrepancies between measurements of circulating citrullinated histones and other markers and with those of tissues [31].

Also, neutrophils can generate markers such as cfDNA and CitH3 by mechanisms other than NETosis [2, 12]. Therefore, the use of citrullinated histones (CitH3) as specific NET markers, despite frequent employment, is controversial [35, 37]. Lastly, markers with the potential to detect NETs have not been designed, and it is unclear whether NET markers are drivers of disease severity, a simple consequence of acute inflammation [2], or both.

The clear association of cfDNA, neutrophil counts, and IL-8 with severity and mortality, and the (much poorer) relationship of the more specific markers of NETosis suggest an important role for neutrophils in severe COVID-19 cases [30, 41]; however, it is difficult to establish a clear relationship between circulating NET markers and these outcomes. The strong correlation of the 2 markers considered the more specific for NETs, MPO-DNA and NE-DNA, in all severity groups and throughout hospitalization, and the very limited correlation of the remaining biomarkers with each other, suggest that MPO-DNA and NE-DNA are the best circulating NETosis markers. Overall, the results of our study suggest, like others [2, 12, 42, 43], that cfDNA is not a marker with high specificity for NETosis, because it is also released by various hematopoietic cells and from a wide range of tissues after cell destruction, as has been demonstrated in COVID-19 [19, 34, 42]. Also, cfDNA is a DAMP, capable of amplifying the inflammatory response [44, 45] via toll-like receptor 9 (TLR-9) [44]. NET formation is likely better reflected by more neutrophil-specific markers, such as MPO-DNA and NE-DNA, whereas cfDNA better reflects cellular damage in a broader sense, correlating with disease severity parameters and better predicting disease outcomes [2, 12]. The widespread inclusion of cfDNA in previous studies could have overestimated the role of NETs in COVID-19 severity. The relationship detected between cfDNA and sex (p = 0.002) only in the COVID group (not in the controls) supports that males have a higher immune response and tissue damage than females, as has been previously reported [19].

On days 10–16, when viral control appears to be achieved in those with a good prognosis, the significantly higher blood levels of MPO-DNA and NE-DNA complexes in those who died suggest increased NET production, concurrent with a lack of viral clearance in the second week of evolution [46], associated with poor prognoses [47]. Our results suggest that NET status varies at different time points in the evolution of COVID-19; that an increase in NET release during the second week of disease progression, probably associated with a higher viral load, can contribute to poorer outcomes; and that NET detection during this period could predict its evolution. An association between NETs and COVID-19 severity could encourage new treatment approaches, such as DNases [25], recombinant human DNase-I [48], IL-8 receptor antagonists [30], certain monoclonal antibodies, PAD4 or nicotinamide adenine dinucleotide phosphate (NADPH) inhibitors [49], Fostamatinib [50], Resolvin T-series [51], and current and novel histone inhibitors [36].

Our study has limitations. Our cohort was small, from a single hospital. We were unable to measure viral load and thus demonstrate a parallel association of increased and sustained viral load and NET formation on days 10–16 in patients with poor prognosis. Quantification problems for NETosis and cytokines have been mentioned. The strengths of our study include that there is scarce information on NETosis markers on patients with COVID-19 progression. As far as we know, ours is the first study systematically including 3 evolutive phases, including the NET biomarker most used in previous studies and 3 markers of those considered more specific combined. Our controls were not healthy, as in most studies, but had comorbidities similar to those of our patients, which gives greater value to comparisons of their parameters with those of the patients.

## Conclusions

Our study has demonstrated the presence of increased markers of both circulating NETs and neutrophil activation and recruitment at all COVID-19 severity levels, from admission throughout the evolution of hospitalised patients, but suggests differences in specificity and quality of some NETosis markers. Although previous studies have reported that these marker levels could be related to disease severity, at present there is no evidence that SARS-CoV-2 can directly induce NET production, and more information is needed on the possible mechanism by which NETs would promote COVID-19 progression [2, 29]. Our results contradict in some ways some of the other previous results. However, in our previous study with the same samples, AUCs for other markers well known for their association with outcomes in COVID-19, such as IL-6, were validated [19]. It seems clear that, with our current limitations in accurately measuring NETs by circulating markers, it is difficult to reach definitive conclusions about the proper role of NETs in the pathogenesis of COVID-19.

### Supplementary Information

**Additional file 1: Table S1**. Demographics and comorbidities of the patients and controls

**Additional file 2: Table S2**: Detailed view of the multivariate model for severity, mortality, and need for ICU.

**Additional file 3: Table S3**. Relationship between NET markers and sex, age, and obesity separately in patients and controls.

**Additional file 4: Table S4**. Sex-biased analysis of NET markers and mortality in total samples.

**Additional file 5: Table S5**. Correlations between usual clinical parameters of severity, neutrophil counts, neutrophil associated cytokines (IL-8 and G-CSF), and NET markers

## Data Availability

The datasets supporting the conclusions of this article are available in the Zenodo repository, in https://zenodo.org/record/8205103 [52].

## Abbreviations

CCDC score: Chinese Center for Disease Control and Prevention classification
cfDNA: Cell-free DNA
CI: Critically ill
CitH3: Citrullinated histone 3
CitH3-NE: Citrullinated histone 3-neutrophil elastase complexes
CitH4-DNA: Citrullinated histone 4-DNA complexes
DAMPs: Damage-associated molecular patterns
GCSF: Granulocyte colony-stimulating factor
H3: Histone 3
IL-1: Interleukin 1
IL-8: Interleukin 8
MI: Moderately ill
MPO-DNA: Myeloperoxidase-DNA complexes
NADPH: Nicotinamide adenine dinucleotide phosphate
NE-DNA: Neutrophilic elastase-DNA complexes
NETs: Neutrophil extracellular traps
PAD4: Peptidylarginine deiminase
ROS: Reactive oxygen species
SI: Severely ill
TLR-9: Toll like receptor 9
WHO OS: World Health Organization ordinal scale
