# Supplementary material for: What is the actual relationship between neutrophil extracellular traps and COVID-19 severity? A longitudinal study
Source: Respir Res. 2024 Jan 19;25:48. doi: 10.1186/s12931-023-02650-9 (PMC10797938; doi:10.1186/s12931-023-02650-9)
Supplement: Supplementary file 1 — Additional file 1: Table S1. Demographics and comorbidities of the patients and controls [file 12931_2023_2650_MOESM1_ESM.docx]

Additional file 1

Additional Table 1

|  | | **Patients**  **N=93** | **Controls**  **N=55** | ***p-value*** |
| --- | --- | --- | --- | --- |
| **Sex** | **Female** | 51 (54.84%) | 30 (54.55%) | 1.000 |
|  | **Male** | 42 (45.27%) | 25 (45.45%) |  |
| **Age (years)** | | 69.00 [55.00; 78.00] | 71.00 [57.00; 79.00] | 0.441 |
| **Hypertension** | | 45 (48.39%) | 17 (30.91%) | 0.056 |
| **Diabetes** | | 28 (30.11%) | 12 (21.82%) | 0.365 |
| **Obesity** | | 13 (13.98%) | 4 (7.27%) | 0.332 |
| **Heart disease** | | 14 (15.05%) | 6 (10.91%) | 0.643 |
| **Chronic respiratory disease** | | 16 (17.20%) | 3 (5.45%) | 0.070 |
| **Liver disease** | | 3 (3.23%) | 0 (0.00%) | 0.295 |
| **Renal disease** | | 8 (8.60%) | 6 (10.91%) | 0.863 |
| **Dementia** | | 18 (19.35%) | 4 (7.27%) | 0.079 |
| **Dyslipidemia** | | 17 (18.28%) | 17 (30.91%) | 0.118 |

Table S1. Demographics and comorbidities of the patients and controls
