# Supplementary material for: What is the actual relationship between neutrophil extracellular traps and COVID-19 severity? A longitudinal study
Source: Respir Res. 2024 Jan 19;25:48. doi: 10.1186/s12931-023-02650-9 (PMC10797938; doi:10.1186/s12931-023-02650-9)
Supplement: Supplementary file 2 — Additional file 2: Table S2: Detailed view of the multivariate model for severity, mortality, and need for ICU. [file 12931_2023_2650_MOESM2_ESM.docx]

Additional file 2

Additional table 2

| **CCDC score** | **Moderate and Severe** | **Critical** | **OR (univariate)** | **OR (multivariate)** |
| --- | --- | --- | --- | --- |
| Age>60  n (%) | 37 (61.7) | 23 (38.3) | 4.51 (1.53-16.66, p=0.012) | 2.70 (0.63-14.33, p=0.201) |
| Male sex  n (%) | 25 (59.5) | 17 (40.5) | 2.79 (1.12-7.24, p=0.030) | 2.51 (0.60-12.03, p=0.216) |
| Hypertension  n (%) | 29 (64.4) | 16 (35.6) | 1.86 (0.75-4.70, p=0.182) | - |
| **Obesity**  **n (%)** | **5 (38.5)** | **8 (61.5)** | **5.14 (1.53-18.82, p=0.009)** | **9.25 (1.54-67.46, p=0.019)** |
| Corticosteroid therapy n (%) | 28 (59.6) | 19 (40.4) | 3.22 (1.27-8.81, p=0.0171) | 1.76 (0.49-6.64, p=0.385) |
| Diabetes  n (%) | 21 (75.0) | 7 (25.0) | 0.75 (0.26-1.99, p=0.575) | - |
| IL-8  Mean (SD) | 69.2 (103.7) | 75.2 (50.5) | 1.04 (1.00-1.08, p=0.051) | - |
| G-CSF  Mean (SD) | 184.2 (312.6) | 226.7 (283.0) | 1.00 (1.00-1.00, p=0.569) | - |
| cfDNA  Mean (SD) | 8.0 (6.1) | 17.4 (14.1) | 1.12 (1.05-1.20, p=0.001) | 1.07 (1.01-1.16, p=0.055) |
| MPO-DNA  Mean (SD) | 0.9 (0.5) | 1.1 (0.8) | 1.52 (0.73-3.29, p=0.250) | - |
| NE-DNA  Mean (SD) | 1.1 (0.4) | 1.3 (0.6) | 1.72 (0.64-4.79, p=0.267) | - |
| CitH3  Mean (SD) | 29.2 (23.0) | 27.3 (30.1) | 1.00 (0.98-1.02, p=0.748) | - |
| **Death** | **No** | **Yes** | **OR (univariate)** | **OR (multivariate)** |
| Age>60  n (%) | 43 (71.7) | 17 (28.3) | 6.13 (1.60-40.44, p=0.021) | 3.17 (0.58-27.61, p=0.223) |
| Male sex  n (%) | 31 (73.8) | 11 (26.2) | 1.91 (0.69-5.46, p=0.215) | 1.10 (0.26-4.62, p=0.891) |
| Hypertension  n (%) | 31 (68.9) | 14 (31.1) | 3.88 (1.33-13.06, p=0.018) | 2.87 (0.68-15.24, p=0.175) |
| Obesity  n (%) | 8 (61.5) | 5 (38.5) | 2.95 (0.79-10.27, p=0.092) | 2.25 (0.45-11.24, p=0.314) |
| Corticosteroid therapy n (%) | 35 (74.5) | 12 (25.5) | 1.91 (0.69-5.64, p=0.222) | - |
| Diabetes  n (%) | 25 (89.3) | 3 (10.7) | 0.37 (0.08-1.23, p=0.138) | - |
| IL-8  Mean (SD) | 68.0 (99.3) | 81.6 (55.6) | 1.00 (1.00-1.01, p=0.584) | - |
| G-CSF  Mean (SD) | 182.8 (298.7) | 246.1 (326.1) | 1.00 (1.00-1.00, p=0.454) | - |
| **cfDNA**  **Mean (SD)** | **8.8 (8.0)** | **17.5 (13.1)** | **1.09 (1.03-1.16, p=0.006)** | **1.08 (1.02-1.15, p=0.014)** |
| MPO-DNA  Mean (SD) | 0.9 (0.5) | 1.1 (0.9) | 1.56 (0.71-3.35, p=0.234 | - |
| NE-DNA  Mean (SD) | 1.1 (0.5) | 1.3 (0.6) | 1.80 (0.63-5.11, p=0.246) | - |
| CitH3  Mean (SD) | 30.0 (24.7) | 23.6 (26.1) | 0.99 (0.96-1.01, p=0.335) | - |
| **ICU admittance** | **No** | **Yes** | **OR (univariate)** | **OR (multivariate)** |
| Age>60  n (%) | 51 (85.0) | 9 (15.0) | 1.76 (0.48-8.41, p=0.421) | 0.69 (0.05-10.85, p=0.780) |
| Male sex  n (%) | 34 (85.0) | 8 (19.0) | 2.76 (0.80-11.06, p=0.119) | 1.84 (0.08-63.05, p=0.695) |
| Hypertension  n (%) | 40 (88.9) | 5 (11.1) | 0.73 (0.20-2.48, p=0.619) | - |
| **Obesity**  **n (%)** | **7 (53.8)** | **6 (46.2)** | **10.57 (2.69-43.67, p=0.001)** | **46.21 (3.76-2138.71, p=0.011)** |
| Corticosteroid therapy n (%) | 36 (76.6) | 11 (23.4) | 13.75 (2.50-257.24, p=0.014 | 19.62 (1.07-2584.67, p=0.114) |
| Diabetes  n (%) | 25 (89.3) | 3 (10.7) | 0.75 (0.16-2.75, p=0.680) | - |
| IL-8  Mean (SD) | 71.3 (97.6) | 67.9 (32.7) | 1.00 (0.99-1.01, p=0.909) | - |
| G-CSF  Mean (SD) | 199.5 (324.7) | 170.9 (41.8) | 1.00 (0.99-1.00, p=0.775) | - |
| **cfDNA**  **Mean (SD)** | **8.7 (6.5)** | **23.7 (17.4)** | **1.14 (1.06-1.25, p=0.001)** | **1.22 (1.07-1.53, p=0.025)** |
| MPO-DNA  Mean (SD) | 0.9 (0.6) | 1.1 (0.8) | 1.57 (0.61-3.46, p=0.272) | - |
| NE-DNA  Mean (SD) | 1.1 (0.4) | 1.4 (0.7) | 2.49 (0.78-7.95, p=0.100) | - |
| CitH3  Mean (SD) | 30.1 (25.8) | 18.8 (15.3) | 0.97 (0.92-1.00, p=0.174) | - |

Table S2. Detailed view of the multivariate model for severity, mortality, and need for ICU.
