# Supplementary material for: What is the actual relationship between neutrophil extracellular traps and COVID-19 severity? A longitudinal study
Source: Respir Res. 2024 Jan 19;25:48. doi: 10.1186/s12931-023-02650-9 (PMC10797938; doi:10.1186/s12931-023-02650-9)
Supplement: Supplementary file 3 — Additional file 3: Table S3. Relationship between NET markers and sex, age, and obesity separately in patients and controls. [file 12931_2023_2650_MOESM3_ESM.docx]

Additional file 3

Additional table 3

| Univariate model (Patients) | | | | |
| --- | --- | --- | --- | --- |
| Sex |  | **Female** | **Male** | **OR (univariate)** |
| cfDNA | Mean (SD) | 7.5 (5.4) | 14.7 (12.6) | 1.11 (1.04-1.20, **p=0.002**) |
| MPO-DNA | Mean (SD) | 0.9 (0.5) | 1.1 (0.7) | 1.70 (0.82-4.25, p=0.183) |
| NE-DNA | Mean (SD) | 1.1 (0.4) | 1.3 (0.6) | 2.75 (1.01-9.68, p=0.076) |
| CitH3 | Mean (SD) | 25.1 (17.6) | 33.3 (31.7) | 1.01 (1.00-1.03, p=0.139) |
| Univariate model (Patients) | | | | |
| Age>60 years |  | **>60 years** | **<60 years** | **OR (univariate)** |
| cfDNA | Mean (SD) | 12.1 (9.6) | 8.1 (9.9) | 0.95 (0.88-1.00, p=0.082) |
| MPO-DNA | Mean (SD) | 1.0 (0.6) | 0.9 (0.6) | 0.66 (0.23-1.41, p=0.342) |
| NE-DNA | Mean (SD) | 1.2 (0.5) | 1.1 (0.4) | 0.63 (0.19-1.68, p=0.402) |
| CitH3 | Mean (SD) | 27.6 (24.4) | 30.5 (26.1) | 1.00 (0.99-1.02, p=0.595) |
| Univariate model (Patients) | | | | |
| Obesity |  | **No** | **Yes** | **OR (univariate)** |
| cfDNA | Mean (SD) | 10.4 (9.8) | 11.7 (10.3) | 1.01 (0.95-1.07, p=0.668) |
| MPO-DNA | Mean (SD) | 1.0 (0.7) | 0.9 (0.3) | 0.92 (0.24-2.18, p=0.876) |
| NE-DNA | Mean (SD) | 1.1 (0.4) | 1.4 (0.6) | 2.29 (0.77-6.99, p=0.119) |
| CitH3 | Mean (SD) | 30.1 (26.2) | 20.4 (13.8) | 0.98 (0.94-1.01, p=0.205) |
| Univariate model (Controls) | | | | |
| Sex |  | **Female** | **Male** | **OR (univariate)** |
| cfDNA | Mean (SD) | 3.33 (2.27) | 2.76 (1.75) | 1.00 (1.00-1.00, p=0.317) |
| MPO-DNA | Mean (SD) | 0.7 (0.5) | 0.8 (0.7) | 1.24 (0.47-3.63, p=0.657) |
| NE-DNA | Mean (SD) | 0.9 (0.6) | 0.9 (0.5) | 1.07 (0.38-3.03, p=0.896) |
| CitH3 | Mean (SD) | 31.2 (20.7) | 23.2 (14.3) | 0.97 (0.94-1.00, p=0.117) |

Table S3. Table S3. Relationship between NET markers and sex, age, and obesity separately in patients and between NET markers and sex in controls
