# Supplementary material for: What is the actual relationship between neutrophil extracellular traps and COVID-19 severity? A longitudinal study
Source: Respir Res. 2024 Jan 19;25:48. doi: 10.1186/s12931-023-02650-9 (PMC10797938; doi:10.1186/s12931-023-02650-9)
Supplement: Supplementary file 4 — Additional file 4: Table S4. Sex-biased analysis of NET markers and mortality in total samples. [file 12931_2023_2650_MOESM4_ESM.docx]

Additional file 4

Additional table 4

| FEMALES | | | | |
| --- | --- | --- | --- | --- |
|  | **[ALL]** | **Survivors** | **Non-survivors** | **p-value** |
|  | *N=110* | *N=91* | *N=19* |  |
| MPO-DNA | 0.76 [0.63;0.96] | 0.77 [0.63;0.96] | 0.75 [0.69;0.87] | 0.763 |
| NE-DNA | 1.04 [0.89;1.18] | 1.04 [0.89;1.18] | 0.95 [0.89;1.11] | 0.491 |
| cfDNA | 6.78 [4.12;10.35] | 5.98 [3.52;8.92] | 14.49 [7.15;21.96] | <0.001 |
| CitH3 | 28.56 [19.44;54.89] | 28.56 [20.58;53.77] | 28.99 [13.66;75.75] | 0.870 |

| males | | | | |
| --- | --- | --- | --- | --- |
|  | **[ALL]** | **Survivors** | **Non-survivors** | **p-value** |
|  | *N=91* | *N=67* | *N=24* |  |
| MPO-DNA | 0.88 [0.64;1.10] | 0.87 [0.63;1.08] | 0.94 [0.71;1.74] | 0.277 |
| NE-DNA | 1.06 [0.87;1.37] | 1.05 [0.85;1.33] | 1.25 [0.97;1.66] | 0.116 |
| cfDNA | 10.16 [5.99;18.91] | 9.74 [5.10;15.77] | 17.92 [8.01;31.72] | 0.005 |
| CitH3 | 27.45 [14.23;51.99] | 26.37 [16.84;53.46] | 30.03 [10.97;45.53] | 0.625 |

Table S4. Sex-biased analysis of NET markers and mortality in total samples.
