# Supplementary material for: What is the actual relationship between neutrophil extracellular traps and COVID-19 severity? A longitudinal study
Source: Respir Res. 2024 Jan 19;25:48. doi: 10.1186/s12931-023-02650-9 (PMC10797938; doi:10.1186/s12931-023-02650-9)
Supplement: Supplementary file 5 — Additional file 5: Table S5. Correlations between usual clinical parameters of severity, neutrophil counts, neutrophil associated cytokines (IL-8 and G-CSF), and NET markers [file 12931_2023_2650_MOESM5_ESM.docx]

Additional file 5

Additional table 5

| Total samples (n=201) | **Leukocytes/mm3** | **Neutrophils /mm3** | **Lymphocytes/mm3** | **N/L ratio** | **CRP (mg/L)** | **LDH (U/L)** | **IL-8 (pg/mL)** | **G-CSF (pg/mL)** | **MPO-DNA** | **NE-DNA** | **cfDNA (ng/mL)** | **CitH3 (ng/mL)** |
| --- | --- | --- | --- | --- | --- | --- | --- | --- | --- | --- | --- | --- |
| Leukocytes/mm3 | --- | r= 0.517  p<0.001 | r= -0.081  p=0.251 | r= 0.462  p<0.001 | r= 0.437  p<0.001 | r= 0.144  p=0.055 | r= 0.217  p<0.001 | r= 0.184  p=0.011 | r= 0.016  p=0.831 | r= 0.054  p=0.460 | r= 0.472  p<0.001 | r= 0.322  p<0.001 |
| Neutrophils/mm3 | r= 0.517  p<0.001 | --- | r= -0.164  p=0.020 | r= 0.646  p<0.001 | r= 0.371  p<0.001 | r= 0.208  p=0.005 | r= 0.074  p=0.309 | r= 0.078  p=0.285 | r= 0.049  p=0.514 | r= 0.174  p=0.017 | r= 0.266  p<0.001 | r= 0.086  p=0.236 |
| Lymphocytes/mm3 | r= -0.081  p=0.251 | r= -0.164  p=0.020 | --- | r= -0.460  p<0.001 | r= -0.323  p<0.001 | r= -0.339  p<0.001 | r= 0.009  p=0.906 | r= -0.153  p=0.034 | r= 0.080  p=0.281 | r= 0.113  p=0.124 | r= -0.329  p<0.001 | r= -0.023  p=0.755 |
| N/L ratio | r= 0.462  p<0.001 | r= 0.646  p<0.001 | r= -0.460  p<0.001 | --- | r= 0.457  p<0.001 | r= 0.254  p=0.001 | r= 0.046  p=0.524 | r= 0.003  p=0.021 | r= 0.095  p=0.965 | r= 0.054  p=0.199 | r= 0.473  p<0.001 | r= 0.118  p=0.106 |
| CRP (mg/L) | r= 0.437  p<0.001 | r= 0.371  p<0.001 | r= -0.323  p<0.001 | r= 0.457  p<0.001 | --- | r= 0.290  p<0.001 | r= -0.025  p=0.742 | r= 0.141  p=0.060 | r= -0.047  p=0.541 | r= -0.057  p=0.455 | r= 0.440  p<0.001 | r= -0.006  p=0.937 |
| LDH (U/L) | r= 0.144  p=0.055 | r= 0.208  p=0.005 | r= -0.339  p<0.001 | r= 0.254  p=0.001 | r= 0.290  p<0.001 | --- | r= -0.069  p=0.369 | r= 0.056  p=0.468 | r= -0.022  p=0.784 | r= 0.011  p=0.892 | r= 0.256  p=0.001 | r= -0.125  p=0.106 |
| IL-8 (pg/mL) | r= 0.217  p<0.001 | r= 0.074  p=0.309 | r= 0.009  p=0.906 | r= 0.046  p=0.524 | r= -0.025  p=0.742 | r= -0.069  p=0.369 | --- | r= 0.042  p=0.563 | r= 0.405  p<0.001 | r= 0.079  p=0.281 | r= 0.165  p=0.023 | r= -0.349  p<0.001 |
| G-CSF (pg/mL) | r= 0.184  p=0.011 | r= 0.078  p=0.285 | r= -0.153  p=0.034 | r= 0.003  p=0.021 | r= 0.141  p=0.060 | r= 0.056  p=0.468 | r= 0.042  p=0.563 | --- | r= -0.001  p=0.988 | r= -0.047  p=0.532 | r= 0.077  p=0.304 | r= -0.052  p=0.490 |
| MPO-DNA | r= 0.016  p=0.831 | r= 0.049  p=0.514 | r= 0.080  p=0.281 | r= 0.095  p=0.965 | r= -0.047  p=0.541 | r= -0.022  p=0.784 | r= 0.405  p<0.001 | r= -0.001  p=0.988 | --- | r= 0.483  p<0.001 | r= 0.095  p=0.203 | r= 0.019  p=0.797 |
| NE-DNA | r= 0.054  p=0.460 | r= 0.174  p=0.017 | r= 0.113  p=0.124 | r= 0.054  p=0.199 | r= -0.057  p=0.455 | r= 0.011  p=0.892 | r= 0.079  p=0.281 | r= -0.047  p=0.532 | r= 0.483  p<0.001 | --- | r= 0.168  p=0.022 | r= -0.010  p=0.891 |
| cfDNA (ng/mL) | r= 0.472  p<0.001 | r= 0.266  p<0.001 | r= -0.329  p<0.001 | r= 0.473  p<0.001 | r= 0.440  p<0.001 | r= 0.256  p=0.001 | r= 0.165  p=0.023 | r= 0.077  p=0.304 | r= 0.095  p=0.203 | r= 0.168  p=0.022 | --- | r= 0.277  p<0.001 |
| CitH3 (ng/mL) | r= 0.322  p<0.001 | r= 0.086  p=0.236 | r= -0.023  p=0.755 | r= 0.118  p=0.106 | r= -0.006  p=0.937 | r= -0.125  p=0.106 | r= -0.349  p<0.001 | r= -0.052  p=0.490 | r= 0.019  p=0.797 | r= -0.010  p=0.891 | r= 0.277  p<0.001 | --- |

Table S5. Correlations between usual clinical parameters of severity, neutrophil counts, neutrophil associated cytokines (IL-8 and G-CSF), and NET markers (MPO-DNA and NE-DNA complex, cfDNA and CitH3) in all samples (n=201)
